# Chest X‐Ray Interpretation in the EPICO Pediatric COVID‐19 Study: Addressing Misconceptions

**DOI:** 10.1002/ppul.71274

**Published:** 2025-09-09

**Authors:** Luis Miguel Navarro‐Ramirez, Pablo Vásquez‐Hoyos, María Lucía Mesa‐Rubio, Melisa Naranjo Vanegas, Daniela Duarte‐Montero, Claudia Burgos, Laura Melissa Mendez, María Margarita Rodriguez, Arianna Martinez, Paola Sanchez, Carolina Tovar, Gabriela Friedrich, Gustavo Adolfo Triana‐Rodriguez, Mónica Royero‐Arias, Jessica Echeverry, Tamara Gamo, Luz Ángela Moreno, Olga Lucía Baquero, Luz Marina Mejía, Sonia Restrepo‐Gualteros, Sergio Moreno‐Lopez, Juan Gabriel Piñeros, Carlos Álvarez‐Moreno, Alejandro Díaz‐Díaz, Iván Felipe Gutierrez, Clara Galvis‐Diaz, José Manuel Nieto, Irati Gastesi, Cinta Moraleda, Alfredo Tagarro García, Andrea Ramirez Varela, EPICO‐Colombia and EPICO‐AEP Working Groups

**Affiliations:** ^1^ School of Medicine Universidad de los Andes Bogotá D.C. Colombia; ^2^ Pediatric Clinical Research Center, Department of Pediatrics, Rutgers Robert Wood Johnson Medical School Rutgers University New Brunswick New Jersey USA; ^3^ Red Colaborativa Pediátrica de Latinoamérica (LARed Network) Montevideo Uruguay; ^4^ School of Medicine Universidad Nacional de Colombia Bogotá D.C. Colombia; ^5^ Department of Pediatrics, Fundación Santa Fe de Bogotá Universidad de los Andes Bogotá D.C. Colombia; ^6^ Medical Imaging & AI Group ‐ Bioscience Center Ayudas Diagnósticas Sura Medellín Antioquia Colombia; ^7^ PediAFe Research Group, Fundación Santa Fe de Bogotá Universidad de los Andes Bogotá D.C. Colombia; ^8^ Department of Diagnostic Imaging Fundación Santa Fe de Bogotá Bogotá D.C. Colombia; ^9^ Pediatric Radiology Servicios de Salud San Vicente Fundación Medellín Antioquia Colombia; ^10^ HOMI, Fundación Hospital Infantil de la Misericordia Bogotá D.C. Colombia; ^11^ Clínica Infantil Colsubsidio Bogotá D.C. Colombia; ^12^ Instituto de Ortopedia Infantil Roosevelt Bogotá D.C. Colombia; ^13^ Clínica Universitaria Colombia, Clínica Colsanitas Bogotá D.C. Colombia; ^14^ Pediatric Infectious Diseases, Hospital Pablo Tobón Uribe Medellín Antioquia Colombia; ^15^ Pediatric Infectious Diseases, Hospital General de Medellín Medellín Antioquia Colombia; ^16^ Pediatric Infectious Diseases, Clínica Infantil Santa Maria del Lago, Clínica Colsanitas Bogotá D.C. Colombia; ^17^ Hospital Militar Central Bogotá D.C. Colombia; ^18^ Hospital Regional de la Orinoquía Yopal Casanare Colombia; ^19^ Fundación Investigación Biomédica Hospital Universitario 12 de Octubre (FIBH12O), Instituto de Investigación Hospital 12 de Octubre (imas12) Madrid Spain; ^20^ Department of Epidemiology School of Public Health, The University of Texas Health Science Center at Houston Houston Texas USA; ^21^ Department of Pediatrics McGovern Medical School, University of Texas Health Sciences Center at Houston (UTHealth) Houston Texas USA; ^22^ School of Public Health, Center for Health Equity, University of Texas Health Science Center at Houston Houston Texas USA

To the Editor,

We appreciate the opportunity to respond to the commentary by Daungsupawong and Wiwanitkit regarding our manuscript, *“What to Look for in Chest X‐Rays of Pediatric Patients With COVID‐19: Insights From a Colombian Cohort”* [1, 2]. We thank the authors for their interest in our work and their thoughtful engagement. Several of the concerns raised in the commentary were, in fact, addressed in the original manuscript; nonetheless, we welcome the opportunity to clarify these points and provide additional context where needed.

## 1 Patient Selection and Generalizability

We agree that clearly defining the study population is essential. However, the statement that “the bulk of the patients were newborns (42%)” is not accurate. According to the *Nelson Textbook of Pediatrics* (22nd ed.), newborns are defined as individuals younger than 28 days [3]. Our study included only children older than 29 days, with most participants being infants. This age distribution aligns with the demographic profile of pediatric COVID‐19 hospitalizations in Colombia during the study period and is consistent with typical patterns observed in pediatric respiratory viral infections [4, 5].

## 2 Influence of Treatment on Radiographic Findings

We recognize that medical treatment may influence radiographic appearances. Accordingly, our analysis included several treatment‐related variables such as oxygen therapy, high‐flow nasal cannula, antibiotics, corticosteroids, mechanical ventilation, ICU admission, and length of hospital stay. Although certain findings, such as alveolar opacities and consolidation, demonstrated higher interobserver agreement, they did not consistently correlate with clinical severity. These results highlight the limited utility of chest radiographs as independent tools for assessing disease progression.

## 3 Interobserver Variability

We agree that variability among radiologists is an important challenge in pediatric imaging. For this reason, our study explicitly assessed interobserver agreement by involving multiple radiologists and reporting kappa coefficients for key findings. As noted, agreement was low for peribronchial thickening (*κ* = 0.1), and moderate for alveolar opacities (*κ* = 0.5) and consolidation (*κ* = 0.4). These findings were discussed in the manuscript and highlight the need for standardized definitions and improved training to enhance consistency in radiographic interpretation. Far from undermining the findings, this variability underscores the relevance of our study in identifying areas where harmonization is needed in clinical practice.

## 4 Clinical Utility and Prognostic Value

The study was designed to characterize radiographic findings and explore associations with clinical outcomes, rather than guide treatment decisions or evaluate therapeutic response. Our results showed no consistent correlation between radiographic patterns and disease severity, clinical management, or prognosis. This suggests that chest X‐rays alone provide limited support for clinical decision‐making in pediatric COVID‐19. Further studies—including those with longitudinal follow‐up—may help define their potential role in ongoing patient care.

## 5 Specific Questions Raised

In response to the questions posed in the Commentary:

Do discrepancies in X‐ray interpretation influence treatment?

- Treatment decisions were made in real time by clinical teams based on radiographic readings available at the bedside, which were different from the retrospective interpretations analyzed in our study. Therefore, discrepancies in interpretation did not appear to impact patient management.

Should radiologists receive targeted training for difficult‐to‐interpret findings?

- We agree that improving consistency in the interpretation of certain findings—such as peribronchial thickening—requires clearer definitions and consensus criteria, rather than reflecting a lack of expertise. Efforts to harmonize interpretation frameworks in pediatric imaging are crucial to reduce variability across studies and clinical practice.

Could studying long‐term radiographic outcomes help clarify treatment‐recovery links?

- While our study did not include long‐term imaging follow‐up, we concur that future research in this area could provide valuable insights into the relationship between treatment and radiographic evolution.

## 6 Future Directions and AI Integration

The integration of artificial intelligence (AI) into radiology presents significant potential for reducing interobserver variability and improving diagnostic accuracy. While AI tools were not utilized in the present study, we endorse ongoing research efforts focused on developing and validating such technologies—especially when applied to high‐quality, standardized imaging data sets. Given the exploratory nature of this study, we did not analyze associations between radiographic findings and clinical parameters such as age, comorbidities, or disease severity. This decision was based on the absence of formal predefined hypotheses and the lack of a sample size calculation to support inferential analyses. We therefore encourage future studies to explore these associations in greater depth using well‐powered epidemiological designs that are appropriately structured to test specific hypotheses.

We hope this response clarifies the points raised and contributes to constructive dialog in the field of pediatric imaging. Continued collaboration and critical appraisal remain central to advancing knowledge and improving care for children with respiratory illnesses.

## Author Contributions

**Luis Miguel Navarro‐Ramirez:** conceptualization, writing – original draft, writing – review and editing, project administration. **Pablo Vásquez‐Hoyos:** writing – review and editing, validation, supervision, project administration. **María Lucía Mesa‐Rubio:** validation, supervision, project administration, writing – review and editing, funding acquisition. **Melisa Naranjo Vanegas:** validation. **Daniela Duarte‐Montero:** validation. **Claudia Burgos:** validation, writing – review and editing. **Laura Melissa Mendez:** validation. **María Margarita Rodriguez:** validation. **Arianna Martinez:** validation. **Paola Sanchez:** validation. **Carolina Tovar:** validation, writing – review and editing. **Gabriela Friedrich:** validation. **Gustavo Adolfo Triana‐Rodriguez:** validation. **Mónica Royero‐Arias:** validation, writing – review and editing. **Jessica Echeverry:** validation. **Tamara Gamo:** validation. **Luz Ángela Moreno:** validation. **Olga Lucía Baquero:** validation. **Luz Marina Mejía:** validation. **Sonia Restrepo‐Gualteros:** validation. **Sergio Moreno‐Lopez:** writing – review and editing, validation. **Juan Gabriel Piñeros:** validation. **Carlos Álvarez‐Moreno:** writing – review and editing, validation, funding acquisition. **Alejandro Díaz‐Díaz:** validation. **Iván Felipe Gutierrez:** validation. **Clara Galvis‐Diaz:** validation. **José Manuel Nieto:** validation. **Irati Gastesi:** validation. **Cinta Moraleda:** validation. **Alfredo Tagarro García:** validation. **Andrea Ramirez Varela:** supervision, writing – review and editing, validation, project administration.

## Conflicts of Interest

The authors declare no conflicts of interest.

## Data Availability

Data derived from public domain resources.
